# Translocation through a narrow pore under a pulling force

**DOI:** 10.1038/s41598-019-53935-3

**Published:** 2019-11-29

**Authors:** Mohammadreza Niknam Hamidabad, Rouhollah Haji Abdolvahab

**Affiliations:** Physics Department, Iran University of Science and Technology (IUST), 16846-13114 Tehran, Iran

**Keywords:** Chemical physics, Computational chemistry, Computational science

## Abstract

We employ a three-dimensional molecular dynamics to simulate a driven polymer translocation through a nanopore by applying an external force, for four pore diameters and two external forces. To see the polymer and pore interaction effects on translocation time, we studied nine interaction energies. Moreover, to better understand the simulation results, we investigate polymer center of mass, shape factor and the monomer spatial distribution through the translocation process. Our results reveal that increasing the polymer-pore interaction energy is accompanied by an increase in the translocation time and decrease in the process rate. Furthermore, for pores with greater diameter, the translocation becomes faster. The shape analysis of the polymer indicates that the polymer shape is highly sensitive to the interaction energy. In great interactions, the monomers come close to the pore from both sides. As a result, the translocation becomes fast at first and slows down at last. Overall, it can be concluded that the external force does not play a major role in the shape and distribution of translocated monomers. However, the interaction energy between monomer and nanopore has a major effect especially on the distribution of translocated monomers on the trans side.

## Introduction

Biopolymer translocation through nanopores is a critical and ubiquitous process in both biology and biotechnology. In this regard, extensive and comprehensive studies have been conducted over the past few decades. Undoubtedly, the study of the translocation of a polymer through nanopores can be considered as one of the most active fields of research in the whole soft matter physics^1–8^. The importance of this process, i.e., polymer translocation (PT), is not limited to understanding its physical and biological dimensions such that essential technological applications, including DNA sequencing^9–13^, controlled drug delivery^14,15^, gene therapy^14,16–18^, and biological sensors are available^9,19^.

Moreover, the passage of biopolymers such as DNA and RNA through nuclear pore complexes^20–23^, virus RNA injection into the host cell^24,25^ and passing proteins through the cell organelle membrane channels^3^ are some other biologic processes which have doubled the importance of this issue.

The polymer translocation is a phenomenon which engages with how polymers move from one side of the pore to another side. In the process, the bio-polymer must overcome the entropic barrier^1,26–28^. Hence, applying an external force on the polymer for overcoming the entropic barrier is one of the most common PT methods used both in the laboratory and computational simulations^5,6,29–34^. To deal with this issue, *in vivo* PT driven by assisted proteins called chaperone is proposed^4,35–39^.

In the following simulation, we used the driven polymer translocation through a nanopore, by applying an external force. Generally, in the driven polymer translocation, several parameters (e.g., length and radius of the nanopore, the applied external force, and the friction coefficient of both Cis and Trans environments) have been investigated by previous articles^2,40–43^. However, the interaction energy (i.e., potential depth) between the monomer-nanopore and its effect on the translocation time, has rarely been investigated.

In this paper, we used a coarse-grained molecular dynamics method to simulate the translocation of a driven-polymer through the nanopore under an external force. The simulation includes nine different interactions between polymer and nanopore, four nanopore diameters, and two different external forces.

## Theory and Simulation Details

### System

#### Polymer

In the following 3D simulation, the polymer chain is modeled as a bead-spring chain of Lennard-Jones (LJ) particles^44–46^. The excluded volume interaction between all the monomers is modeled by a short-range repulsive LJ potential:

1 $${U}_{LJ}=\{\begin{array}{ll}4\epsilon [{(\frac{\sigma }{r})}^{12}-{(\frac{\sigma }{r})}^{6}]+\epsilon  & r\le {2}^{\frac{1}{6}}\sigma \\ 0 & {\rm{otherwise}}\end{array}$$

where *σ* is the diameter of each monomer and $\epsilon$ is the potential depth.

For connectivity between adjacent monomers, Finite Extension Nonlinear Elastic (FENE) potential was used:

2 $${U}_{FENE}=-\,\frac{1}{2}k{R}_{0}^{2}\,\mathrm{ln}\,(1-\frac{{r}^{2}}{{R}_{0}^{2}}).$$

where r is the distance between two adjacent monomers, k and *R*_0_ are the spring constant and the maximal stretching length for adjacent monomers respectively.

For other interactions, such as monomer-wall and monomer-nanopore, also the LJ potential is used. However, by changing the *r*_*cut*_ from ${2}^{\frac{1}{6}}\sigma$ to 2.5*σ*, the LJ interaction changes from purely repulsive (monomer-monomer and monomer-wall) interactions to repulsive and attractive (monomer-nanopore) interaction.

For interaction between polymer and nanopore, the following LJ potential was used:

3 $${U}_{LJ}=\{\begin{array}{ll}4\epsilon [{(\frac{\sigma }{r})}^{12}-{(\frac{\sigma }{r})}^{6}]+\epsilon  & r\le 2.5\sigma \\ 0 & {\rm{otherwise}}\end{array}$$

Finally, the polymer is composed of 50 identical monomers.

#### Membrane

In present work, the membrane is composed of a 6*σ* long cylindrical nanopore and two walls on each side of the nanopore entrances. Besides, all of them are modeled as continuous shapes which means the distance of monomer to any part of the membrane is the distance of the monomer to the surface of that part. Furthermore, four different nanopore diameters of 3*σ*, 4*σ*, 5*σ*, *and* 6*σ* were used.

#### System methodology

We investigated the dynamics of polymer translocation by the Langevin dynamics (LD) method. In this method, the following equation can be written for each monomer:

4 $$m\ddot{\overrightarrow{r}}={\overrightarrow{F}}_{i}^{C}+{\overrightarrow{F}}_{i}^{F}+{\overrightarrow{F}}_{i}^{R}$$

where m is the monomer mass. Moreover, the ${\overrightarrow{F}}_{i}^{C}$, ${\overrightarrow{F}}_{i}^{F}$, and ${\overrightarrow{F}}_{i}^{R}$ are the conservative, frictional, and the random forces applied on the *i*’*s* monomer, respectively. The frictional forces are connected to the monomer’s speed by the following equation:

5 $${\overrightarrow{F}}_{i}^{F}=-\,\xi {\overrightarrow{V}}_{i}$$

where *ξ* is the frictional coefficient. One also can write for the conservative forces:

6 $${\overrightarrow{F}}_{i}^{C}=-\,\overrightarrow{\nabla }({U}_{LJ}+{U}_{FENE})+{\overrightarrow{F}}_{external}$$

where the last term is the external force, exerted only on the monomers that are inside the nanopore and is defined as:

7 $${\overrightarrow{F}}_{external}=f\hat{x}$$

in which, $\hat{x}$ is a unit vector in the direction along the pore axis and towards the Trans side. Here the external force (pulling force) is exerted only on the monomers within the pore.

### Settings

**Figure 1:**
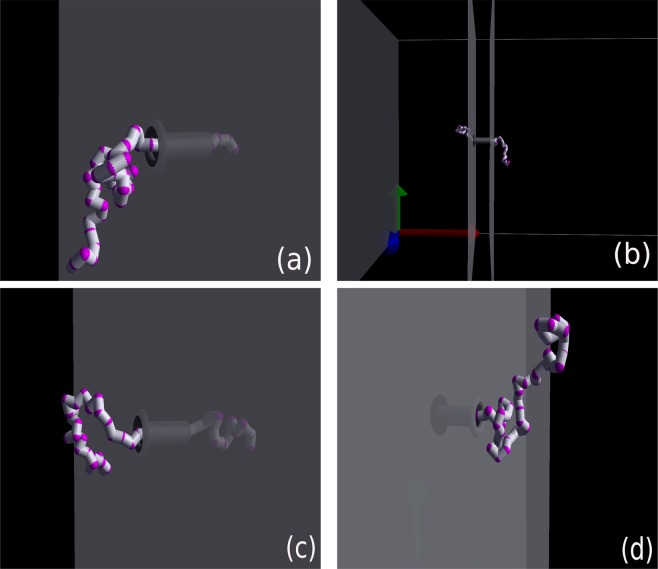
Polymer during a translocation process through the nanopore (**a**) at the beginning of the translocation, (**b**,**c**) in the middle of the translocation and (**d**) at the end of the translocation.

The initial configuration of the system is such that the first monomer is at the end of a nanopore. Then, the remaining monomers have placed close to their equilibrium position relative to each other, and in the front of the nanopore. After placing the monomers, we allow them to achieve their equilibrium as a whole polymer. In the equilibration process, we fix a few monomers in the nanopore and allow the rest of the monomers, i.e., the polymer tail, to move freely until reaching the equilibrium. The equilibration process lasts from about 20% for the slowest up to about 40% for the fastest translocation, of each PT time through the nanopore. Afterward, the process of translocation begins (please see the translocation movie in the supplementary materials). Here, we translocated the polymer for at least 1,500 times to reach a rather good time distribution (Fig. 1)^47^.

To find the equilibrium point, we calculate the radius of gyration of the polymer through the time. The equilibration process continues until the changes in the radius of gyration becomes as small as 2*σ*.

Using *σ*, ${\epsilon }_{0}$ and monomer mass *m*, the energy, length, and mass scales are fixed. So, the time unit of the simulation can be set^36^:

8 $${t}_{LJ}={(\frac{m{\sigma }^{2}}{{\epsilon }_{0}})}^{\frac{1}{2}}$$

The external forces were applied on the monomers inside the pore. We pick the forces from two different regions of strong and medium as the external force in the pore. The relation determining this region for the average force is^29^:

9 $$\frac{{k}_{B}T}{\sigma {N}^{\nu }}\le F\le \frac{{k}_{B}T}{\sigma }$$

where *ν* is the Flory exponent, and *N* stands for the total number of monomers. The magnitude of the strong and medium forces employed in the simulation are $2{\epsilon }_{0}/\sigma$ and $1{\epsilon }_{0}/\sigma$, respectively where ${\epsilon }_{0}$ is defined below (Please note that the *k*_*B*_*T*/*σ* = 1.2, see below).

### Simulation parameters

The parameters in the simulation are the cutoff radius for LJ interactions of the nanopore and the polymer, which is 2.5*σ* while for monomer-monomer and monomer-wall, it is ${2}^{\frac{1}{6}}\sigma$. Considering the persistence length as 7.5 angstroms^48^, the kuhn length *σ* = 1.5 *nm*. Taking the DNA as our polymer, the mass of a bead $m\simeq 936\,amu$ and if we use the energy as the previus works ${k}_{B}T=1.2{\epsilon }_{0}$. We use $\epsilon ={\epsilon }_{0}$ for all the LJ interactions, except the interaction between the polymer and the nanopore which is a multiple of ${\epsilon }_{0}$. For the temperature, *T* = 295 *K* the energy ${\epsilon }_{0}\simeq 3.39\times {10}^{-21}$ and the thus our time units ${t}_{LJ}={(m{\sigma }^{2}/{\epsilon }_{0})}^{\frac{1}{2}}\simeq 32.1\,ps$. Moreover, the friction coefficient is *ξ* = 0.7*m*/*t*_*LJ*_. For the FENE potential, the spring constant is $k=30{\epsilon }_{0}/{\sigma }^{2}$ and *R*_0_ = 1.5*σ*^36,49^. Thus, the force unit is ${\epsilon }_{0}/\sigma$. It means that e.g., the $F=2\sim F=2{\epsilon }_{0}/\sigma$.

## Results and Analysis

**Figure 2:**
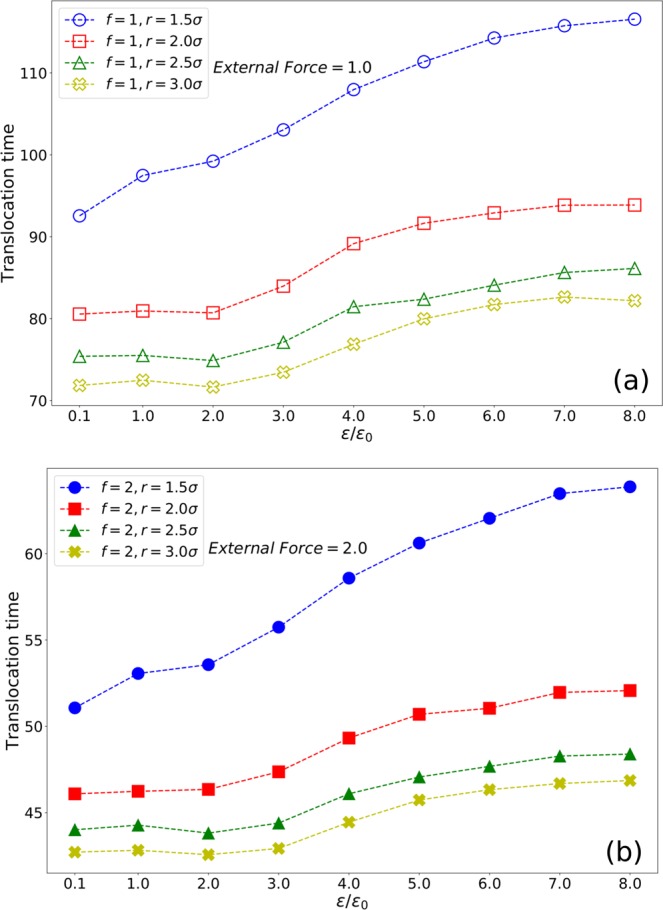
Translocation time versus energy of four different pore diameters under an external force (**a**) *f* = 1 and (**b**) *f* = 2.

Translocation time of the polymer versus polymer and pore interaction energy is plotted in Fig. 2a,b. The interaction energy changes from $\epsilon =0.1$ to $\epsilon =8$. The external force varies from *f* = 1 in Fig. 2a to *f* = 2 in Fig. 2b. As can be seen from both figures, increasing the pore diameter will decrease the translocation time. Moreover, increasing the interaction energy will generally increase the translocation time. For smaller interaction energies ($\epsilon =0.1,1$), due to thermal fluctuations, the increment in translocation time is not expected. As expected, increasing the external force will increase the translocation velocity.

A prominent parameter for describing the dynamics of PT is waiting time. This parameter shows how long it takes for individual monomers to go through the nanopore. To calculate it, we write the times in which the monomer s is just out of the pore in a file. Then the average is calculated. The mean waiting time for the reaction coordinate s is defined as the difference between the averages calculated for monomers s and s + 1.

**Figure 3:**
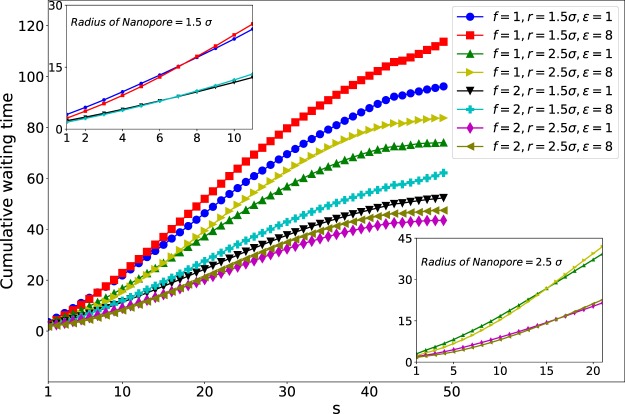
Cumulative waiting times versus s (Note that 50 is the number of monomers, *N*).

**Figure 4:**
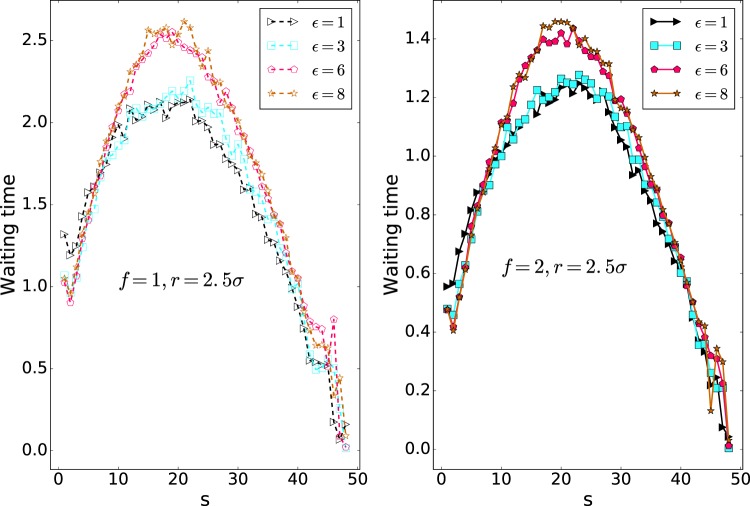
Mean waiting time of the polymer versus monomer number from a nanopore of radius *r* = 2.5*σ*. Note that here 50 is the number of monomers, *N*.

**Figure 5:**
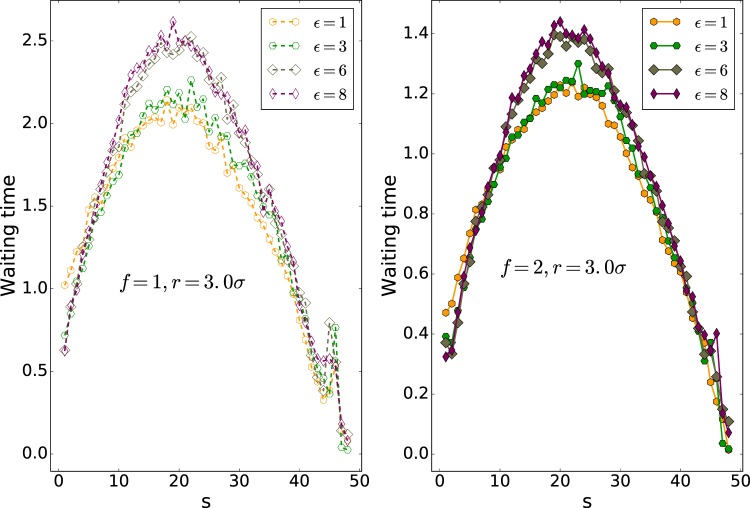
Mean waiting time of the polymer versus monomer number from a nanopore of radius *r* = 3.0*σ*. Note that here 50 is the number of monomers, *N*.

**Figure 6:**
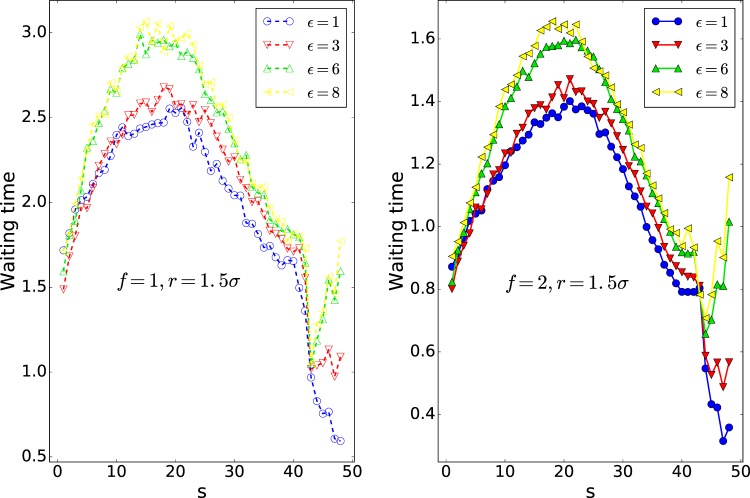
Mean waiting time of the polymer versus monomer number from a nanopore of radius *r* = 1.5*σ*. Note that here 50 is the number of monomers, *N*.

**Figure 7:**
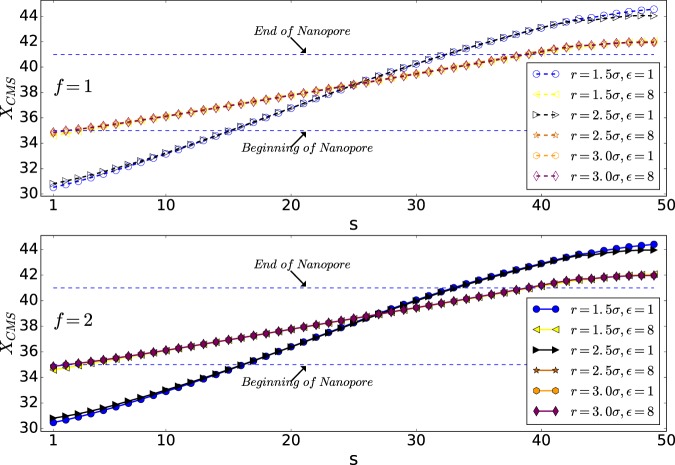
X component of the location of the center of mass (COM) of the polymer versus s. Note that here 50 is the number of monomers, *N*.

The mean waiting time of each monomer for different pore radii of 1.5*σ*, 2.5*σ* and 3.0*σ* is plotted against the monomer number (s), in Figs. 3, 4 and 5, respectively. The maximum of the translocation time is related to the middle monomers due to the entropic barrier of the **Cis** and **Trans** monomers. Thus, the mean waiting plots are nearly bell-shape. The behavior of the final monomers in the interaction energy of ${\epsilon }_{0}=8$ and nanopore of radius *r* = 1.5*σ* is interesting. As shown in Fig. 6, the final monomers waiting times for ${\epsilon }_{0}=8$ and both external forces of *f* = 1 and *f* = 2 are ascending due to crowding effect of monomers on the Trans side and also large interaction energy (see also the Fig. 7). Besides, for the smallest diameter, a higher peak in the mean waiting time can be detected. However, increasing the external force does not have any effect on the general behavior of the mean waiting time.

Cumulative waiting time versus monomer number (s) is presented in Fig. 3. The figure compares interaction energies of ${\epsilon }_{0}=1,8$, external forces of *f* = 1, 2, and pore radii of *r* = 1.5*σ*, 2.5*σ*. Insets are the zoom of the plots at first monomers. As the top inset shows for *r* = 1.5*σ* the polymer with ${\epsilon }_{0}=8$ is faster than the interaction energy of ${\epsilon }_{0}=1$ at 6 first monomers for both forces of *f* = 1, 2. In the wider pore, where *r* = 2.5*σ* the intersection of plots becomes on *s* = 13 (see the low inset of Fig. 3). It means that the high interaction pulls the polymer through the pore and makes it faster, at first, but slows its translocation through the pore in the middle stages. This effect becomes more important as the pore radius becomes larger. This monomer number is expected to rise by increasing the radius of the pore until the point where it is still smaller than the gyration radius of the polymer and also the interaction of the nanopore with the polymer is large enough.

To justify such behaviors in the polymer translocation, we need to look at other parameters such as the center of mass (COM) of the polymer during the passage, the overall shape of the polymer (shape factor), and the spatial distribution of monomers through the translocation process.

Figure 7 shows the X component of the polymer COM versus s. The coordinate of the center of the nanopore is (40, 38, 40). It is of note that the polymer is initially in equilibrium. To discuss the translocation in more details, we focus on *X*_*COM*_, which is the pore direction in Fig. 7. As can be observed, in the first stage of the translocation, the polymers with high interaction energy of ${\epsilon }_{0}=8$ have greater *X*_*COM*_ compared to the polymers with low interaction energy of ${\epsilon }_{0}=1$, suggesting that they reach the equilibrium nearest to the pore as the interaction supports. They are also nearest to the pore in the last stage of the translocation with the same reason. To see the polymer’s behavior in more details, we study the polymer shape using the average aspect ratio *α* and the shape factor *δ*^28,50^. Here, *α* denotes the distribution of the translocated monomers along the pore axis (x) and the plane perpendicular to the pore axis (yz plane), *α* = Δ*x*/(2*r*). Besides, Δ*x* is the maximum of the polymer distance from the pore in the trans side in the x-direction and r is the maximum distance of the polymer from the pore axis (x) in the trans side, $r=\sqrt{{y}_{\max }^{2}+{z}_{\max }^{2}}$ ^46^.

**Figure 8:**
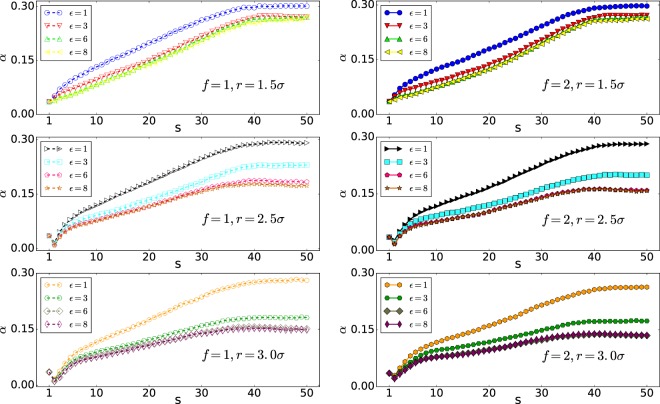
*α* versus s (Note that 50 is the number of monomers, *N*).

As can be seen from Fig. 8, the distribution of monomers in the wider pore of *r* = 2.5*σ* has a smaller value of *α* than the pore with radius *r* = 1.5*σ*, indicating that monomers have distributed widely in the yz-plane. Moreover, it shows that, following the previous discussion, the widest distribution of the monomers is in the case of high interaction energy of ${\epsilon }_{0}=8$ and *r* = 2.5*σ*.

**Figure 9:**
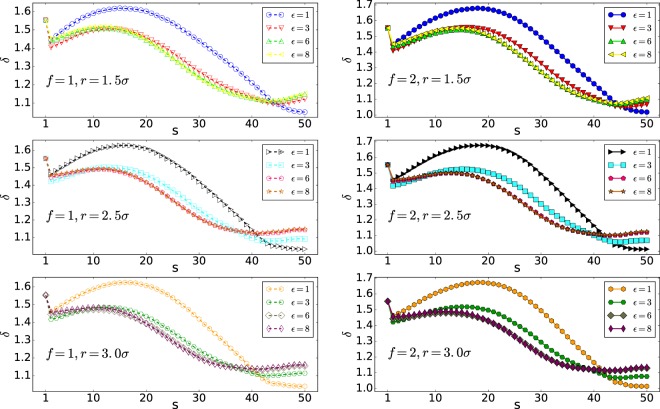
Shape factor *δ* versus s (Note that here 50 is the number of monomers, *N*).

The shape factor *δ* versus s is shown in Fig. 9. This parameter is computed for all the monomers (in both Cis and Trans side) to compare the gyration and the hydrodynamic radius^28^. The upper limit of the shape factor *δ* is for a rod and equals *δ*_*max*_ = 4.0 and the lower limit of it is for a compact sphere and equals *δ* = 0.77^28^.

The results show that increasing the interaction energy will decrease the shape factor variation. Moreover, increasing the external force will increase *δ*, and the polymer becomes more rod shape. In addition, at the final stage of the translocation, the shape factor *δ* will increase by increasing the interaction energy. It means that the polymer with lower interaction energies is more compact concerning those with a higher $\epsilon$.

## Conclusions

We use 3D molecular dynamics to simulate the polymer translocation through a narrow pore driven by an external force. Simulation results show that increasing the polymer-pore interaction energy slows down the translocation (2a, 2b). Moreover, increasing the pore diameter makes the translocation faster, which is in accordance with previous results^51,52^.

The detailed analysis of the polymer shape shows that the polymer tends to reach the pore in high energies at both first and last parts of the translocation process concerning the polymers with lower interaction energies. This causes the translocation of the polymer with higher interaction energy becomes faster at first and slower at last. Moreover, our detailed shape analysis reveals that the polymers with lower energy and in wider pores are less rod shape through the translocation. Also, while the polymer shape is not sensible to the external force (at least in the forces of *f* = 1 and *f* = 2), its shape is very sensitive to the interaction energy between the polymer and nanopore.

Waiting time analysis shows that monomers in the middle of the polymer take more time than others. Also, the monomers in the middle of the polymer have a higher peak for the smallest pore radius, which shows the slowest part of the translocation. In high interaction energy of ${\epsilon }_{0}=8$ and the small pore radius of *r* = 1.5*σ*, the last monomer’s waiting times versus monomer number (s) are ascending. Due to the high interaction and accumulation of the monomers at the trans side, the polymer is not inclined to leave the pore.

In summary, changing the pore diameter and polymer-pore interaction will cause the translocation time, polymer shape through the translocation, accumulation of the monomer at first and last stages of the translocation and waiting time of each monomer to vary widely.

## Appendices

### Meal waiting time

The mean waiting time of each monomer for different pore radii of 2.5*σ* and 3.0*σ* is plotted against the monomer number, s, in Figs. 4 and 5, respectively. The maximum of the translocation time is related to the middle monomers due to the entropic barrier of the cis and trans monomers. As a result, the mean waiting times are bell-shape. Increasing the external force doesn’t have any effect on the general behavior of the mean waiting time.

### Average aspect ratio; *α*

**Figure 10:**
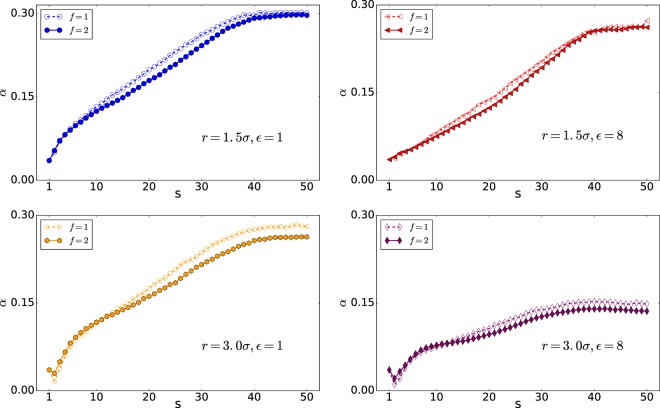
*α* versus monomer number s (Note that here 50 is the number of monomers, *N*).

**Figure 11:**
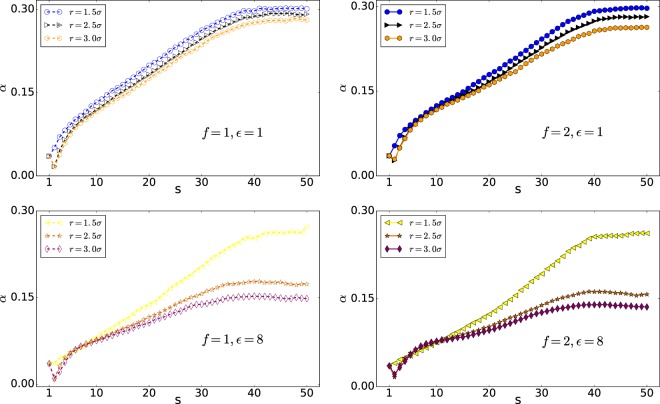
*α* versus monomer number s (Note that here 50 is the number of monomers, *N*).

As the Figs. 10 and 11 show the distribution of monomers in wider pore of *r* = 2.5*σ* has smaller value of *α* than the pore with radius *r* = 1.5*σ* which means monomers have distributed widely in the yz-plane.

### The shape factor *δ*

**Figure 12:**
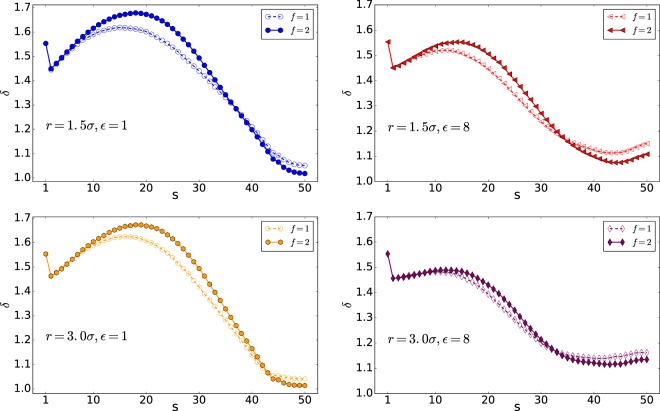
Shape factor *δ* versus monomer number s (Note that here 50 is the number of monomers, *N*).

**Figure 13:**
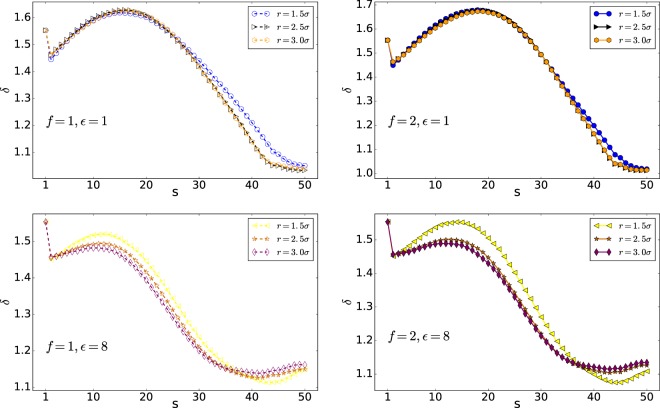
Shape factor *δ* versus monomer number s (Note that here 50 is the number of monomers, *N*).

The shape factor *δ* versus monomer number has been shown in Figs. 12 and 13. This parameter is computed for all the monomers (cis and trans side) and compares the gyration and the hydrodynamic radius^28^. The upper limit of the shape factor *δ* is for a rod and equals *δ*_*max*_ = 4.0 and the lower limit of it is for a compact sphere and equals *δ* = 0.77^28^.

## Supplementary information

Supplementary information

Supplementary information

Supplementary information
